# A case report of peritoneal tuberculosis with multiple miliary peritoneal deposits mimicking advanced ovarian carcinoma

**Published:** 2016

**Authors:** Shahla Yazdani, Mahmod Sadeghi, Abolhasan Alijanpour, Mojgan Naeimi-rad

**Affiliations:** 1 Fateme Zahra Fertility & Infertility Research Health Center, Babol of University of Medical Science Babol, Iran.; 2 Infectious Diseases and Tropical Medicine Research Center, Babol University of Medical Sciences, Babol, Iran.; 3 Department Surgery, Babol University of Medical Sciences, Babol, Iran.; 4 The Clinical Research Development Unit of Rouhani Hospital, Babol University of Medical Sciences, Babol, Iran.

**Keywords:** Tuberculosis, Ovary, CA125, Peritoneal, Carcinoma

## Abstract

**Background:**

Peritoneal tuberculosis accounts 1-2% of all forms of tuberculosis. Peritoneal tuberculosis is an important differential diagnosis for ovarian cancer in women with ascites, adnexal mass and elevated cancer antigen 125 (CA_125_) levels. We report a case of a 32- year -old woman with multiple miliary peritoneal deposits mimicking advanced ovarian carcinoma.

**Case Presentation:**

A 32-year-old drug addicted woman presented with menometrorrhagia, fever and shivering, ascites and pelvis mass. Ultrasonography revealed a 53×65 mm cyst in left ovary and ascites. Multiple miliary peritoneal deposits were observed during laparatomy without any mass, histologic examination confirmed tuberculosis of uterus, tubes, omentum, liver and external surfaces of small intestine. Finally, the patient recovered with anti-tuberculosis treatment.

**Conclusion:**

These findings highlight considering tuberculosis in the differential diagnosis of any patients with adnexal mass, ascitis and elevated serum CA_125_ even with negative cytology and bacteriology test results.

Mycobacterium tuberculosis is the responsible agent of tuberculosis and a major health problem in developing countries (1). Peritoneal tuberculosis is a form of abdominal tuberculosis that involves intestinal tracts, liver, spleen, female genital tract, omentum, parietal and visceral peritoneum (2). This type of tuberculosis accounts 1-2% of all forms of tuberculosis (3). In women, the presence of ascites, adnexal mass and elevated CA_125_ may indicate ovarian cancer but diagnosis of tuberculosis should also be considered in the differential diagnosis (4). This issue is important because it is a treatable condition and develops at age range of 20-40 years, whereas, ovarian cancer occurs in older age groups (5). A large percentage (20.6%) of peritoneal tuberculosis initially present with extra pulmonary (6). 

Peritoneal tuberculosis is a very rare manifestation of tuberculosis with nonspecific presentation of abdominal distension, ascites, tenderness, and fever and weight loss that may result in a significant diagnostic delay, nearly four months (7). In a study from India, 26 patients who underwent laparotomy for ovarian cancer had abdominal- pelvic tuberculosis. They had menstrual dysfunction, abdominal distention, abdominal pain, abdominal mass and elevated CA_125 _(8).The present study presents a case of a 32-year-old woman with multiple miliary peritoneal deposits mimicking advanced ovarian cancer.

## Case Presentation

A 32-year-old-woman G_3_P_2_L_2_ (Gravidity=3, Parity=2, Living child=2) with abdominal pain and abnormal vaginal bleeding referred to Ayatollah Rouhani Hospital in Babol. Pain was localized in the hypogastrium area for 3 months. The patient had menometrorrhagia, fever and shivering, and development ascites and diagnosis of pelvic mass required hospitalization.

 She has been addicted to opium for 5 years and also used methadone. Her vital signs were stable and her lung sounds were clear but sometimes she had a fever of about 38. There was a 53×65 mm cyst in left ovary region in ultrasonography but the ovaries, uterus, liver, kidney and spleen were normal and ascites fluid was reported.

An ovarian malignant mass was reported in spiral CT scan (computerized axial tomography) with contrast. The patient underwent laparotomic surgery in which her uterine tubes were swollen with adhesion in both ovaries with multiple military peritoneal deposits. No mass has been observed in the abdomen and pelvis. The results of histologic examination demonstrated abdominal tuberculosis on her uterus, tubes, omentum, liver and intestinal surface. The results of laboratory test are shown in table 1. After surgery, she received anti-tuberculosis medical treatment using four drugs: *isoniazid, *pyrazinamide, ethambutol hcl and *rifampin.*

**Table 1:** . Results of laboratory parameters in this patient

*WBC*	*5000 u/ml*
*RBC*	*3.82* *×* *10* ^6^ * u/ml*
*Hb*	*9.8 g/dl*
*Hct*	*30.7%*
*AFP*	*NL*
*CA*_125_	*268.9 u/ml*
*CEA*	*NL*
*CA*_19.9_	*NL*
*HIV*	*Negative*
*HBS & HCV Ag*	*Negative*
*TSH*	*3.6 mu/l*

## Discussion

Abdominal tuberculosis with series of clinical signs may mimic ovarian cancer (9). This often leads to unnecessary expensive surgery in women of reproductive age. However most cases can be diagnosed using a laparoscopy (10). CA_125_ can be considered as a marker for the evaluation of treatment response. This patient shows rapid decline in CA_125_ level paralleling clinical response to antituberculosis (11). For preoperative detection of tuberculosis, ascetic fluid adenosine (ADA) and PCR analysis have proven to be useful (12, 13). High level of ADA and the response after an antituberculosis regimen is helpful to avoid invasive diagnostic procedures which are potentially dangerous. In a study of 138 patients suspected to have ovarian malignancies, 5.7% of them showed ovarian tuberculosis after surgery. In these patients, pain and abdominal distension were usual presenting signs (12).

Abnormal menstruation, pain, mass and distension of abdomen were the usual signs of 26 patients with ovarian malignancy diagnosed in a study in India that confirmed abdominal tuberculosis after laparoscopy and histopathology test (8). In addition, frozen section laparoscopy is a non-invasive method in diagnosis and it is suggested during surgery (13). Therefore, the presence of mass in abdomen, ascites and increasing of CA_125_ may suggest the possibility of tuberculosis. 

In conclusion, abdominal tuberculosis should be considered in the differential diagnosis of women with adnexal mass, ascites and elevated CA_125_ even with negative cytology and bacteriological test results. The use of imaging techniques and laparoscopy or finally laparotomy is recommended. In endemic areas, physicians should consider tuberculosis in the differential diagnosis of any case with unusual manifestations.
